# Five-Year Incidence and Progression of Diabetic Retinopathy in Patients with Type II Diabetes in a Tertiary Care Center in Lebanon

**DOI:** 10.1155/2017/9805145

**Published:** 2017-05-31

**Authors:** Carl-Joe Mehanna, Maamoun Abdul Fattah, Hani Tamim, Mona P. Nasrallah, Raya Zreik, Sandra S. Haddad, Jaafar El-Annan, Samih Raad, Randa S. Haddad, Haytham I. S. Salti

**Affiliations:** ^1^Department of Ophthalmology, American University of Beirut Medical Center, Beirut, Lebanon; ^2^Biostatistics Unit, Department of Internal Medicine, American University of Beirut Medical Center, Beirut, Lebanon; ^3^Division of Endocrinology and Metabolism, Department of Internal Medicine, American University of Beirut Medical Center, Beirut, Lebanon; ^4^Fouad Khoury Hospital Bikhazi Medical Group, Beirut, Lebanon; ^5^Department of Ophthalmology, University of Texas Medical Branch, Galveston, TX, USA; ^6^Fellow-Division of Pulmonary, Critical Care & Sleep Medicine, University of Oklahoma Health Sciences Center, USA

## Abstract

**Objective:**

To estimate the 5-year incidence of progression rate and regression rate and risk factors for diabetic retinopathy (DR) in a cohort of Lebanese patients with type II diabetes.

**Methods:**

We followed a cohort of 462 Lebanese patients with type II diabetes for over 5 years at the American University of Beirut Medical Center. Patients underwent yearly complete ophthalmic evaluation and fundus photographs and were assessed for the incidence, stage, and evolution of DR using modified Airlie House classification.

**Results:**

Among the 462 patients, 281 had no DR at baseline. The 5-year cumulative incidence of any DR was 10% (95% CI: 6–13), and only baseline microalbuminuria correlated with the development of DR (OR = 10.53, 95% CI: 4.39–25.23, *p* < 0.0001). Among the 181 patients with baseline DR, the worsening and regression rates of DR were 31.5% (95% CI: 25–38) and 9% (95% CI: 5–13), respectively. Microalbuminuria also approached statistical significance as a risk factor for DR worsening (OR = 1.89, 95% CI: 0.97–3.70, *p* = 0.06).

**Conclusion:**

The 5-year incidence of DR in this hospital-based cohort is relatively low. Microalbuminuria was independently associated with the incidence and progression of the disease. We recommend to screen patients with type II diabetes for microalbuminuria as prognostic for the development and worsening of DR.

## 1. Introduction

Diabetic retinopathy (DR) remains to be a leading cause of visual impairment in the working age population in the Western world today [1]. Available treatment options for visually threatening DR (VTDR) have been suboptimal with regard to restoring vision and preventing further vision loss. Identifying the local incidence of DR, the rates of progression and regression, and more importantly the associated risk factors is essential towards a better understanding of the disease. Modifying such factors when possible might help in preventing or delaying the progression of DR-associated visual impairment. Such epidemiologic numbers and factors have been identified in large scale Western and Far Eastern countries. In fact, duration of diabetes and glycated hemoglobin (HbA_1c_) have been fairly consistently reported as risk factors for the development of DR, with 5- and 10-year incidence rates ranging from 3.9% to 66.9%, respectively [2–6]. Studies about DR incidence in the Eastern Mediterranean region are rare. A study from Scanlon et al. suggested that large differences may exist in DR onset and progression among various populations [7]; these concepts, along with Lebanon having a relatively high prevalence of diabetes [8], lead our group to establish a hospital-based registry of adult patients with diabetes. From this registry, a cohort was put together to look at the epidemiology and microvascular complications of diabetes. Demographic characteristics of this cohort were previously described [9]. Prevalence data and factors associated with the presence of DR in a subset of 560 patients with type II diabetes from the same cohort have similarly been published [10]. After following up this same subset from the cohort for a period of 5 years, our aim was to report the 5-year incidence of DR for this period; in addition, we looked at the rates of progression and regression of DR and their associated risk factors.

## 2. Materials and Methods

### 2.1. Patient Recruitment

This was a prospective cohort study of 560 consecutive patients with diabetes presenting to the diabetes clinic at the American University of Beirut Medical Center (AUBMC) in Beirut, Lebanon, between January 2004 and May 2008. The study was in adherence with the Declaration of Helsinki. After approval of the AUBMC Institutional Review Board, eligible patients were invited to participate in the study. Inclusion and exclusion criteria were reported previously [10]. Briefly, the patients included had type II diabetes, were 18 years or older, agreed to have periodic laboratory tests, and were able to be followed up on a yearly basis. Patients were excluded if they were unable to sign an informed consent form, refused laboratory testing, or were not able to be followed up for an ophthalmologic evaluation on a yearly basis for a period of 5 years.

### 2.2. Ophthalmic Evaluation

Ophthalmic evaluation was done as part of the study on a yearly basis and was independent of the standard ophthalmologic care that the patients were receiving from their primary eye care provider. In fact, it was emphasized to the patients during each encounter that this visit would not replace their regular eye checkup and that they should follow the recommendations of their primary ophthalmologist. The ophthalmic evaluation consisted of a comprehensive eye examination performed by an experienced retina specialist (H. S.) including Snellen best-corrected visual acuity, tonometry, biomicroscopy, and a dilated fundus examination using a 78-diopter Volk aspherical lens.

### 2.3. Retinal Imaging

Digital fundus photographs of the 30° 7 ETDRS fields were taken using the TOPCON TRC-NW6S digital fundus camera (Topcon Corporation, Tokyo, Japan) and stored using the TOPCON IMAGEnet 2000 software. After masking all patient identifiers, images were sent electronically to be evaluated at a remote reading site by another retina specialist (J. E.). In the event of a discrepancy between the fundus photo grading by the two retina specialists, an independent blinded third reader (S. H.) with experience in evaluating DR was asked to referee and resolve the discrepancy. The modified Airlie House classification standard photos were used as a reference for DR changes [11], and the Early Treatment Diabetic Retinopathy Study (ETDRS) score was used to grade the disease [12]. DR was labeled as “present” when there was at least an appearance of a single microaneurysm in at least one eye (minimum ETDRS score level 20). Progression of DR into more advanced levels was defined as a clear worsening of the ETDRS score of at least two levels or more of retinopathy confirmed by both clinical examination and fundus photographs on two consecutive visits. Similarly, regression was defined as a decrease in the DR ETDRS score equivalent to two levels or more or a return to no DR documented by both evaluation techniques on two consecutive visits.

### 2.4. Risk Factor Assessment

Data from both history and chart reviews was collected and included: age, gender, age at diagnosis of diabetes, duration in years since diagnosis of diabetes, family history of diabetes, consanguinity, smoking status and quantity in pack-years, coronary artery disease (CAD), peripheral vascular disease (PVD), cerebrovascular disease (CVD), and neuropathy. Blood pressure (BP), height, weight, and body mass index (BMI) were measured at every visit. Each visit also included taking urine samples for urine micro- and macroalbuminuria and fasting blood tests for lipid profile (total cholesterol, HDL, LDL, and triglyceride levels), serum creatinine, and glycated hemoglobin (HbA_1c_). These were taken and analyzed at the central laboratory of AUBMC using standardized techniques. Albumin-to-creatinine ratio was calculated with 30–299 mg/g designating microalbuminuria. Good glycemic control was defined as HbA_1c_ < 7%.

### 2.5. Statistical Analysis

All data collected was entered into Microsoft Excel 2016 and analyzed using IBM-SPSS; v.23 with statistical significance was set at 5% level. We used descriptive analyses to characterize our cohort and patterns of DR. More specifically, number and percent were for categorical variables, and mean and standard deviation (SD) were used for normally distributed continuous variables, or median and interquartile range (IQR) for non-normally distributed ones. Data normality was assessed using kurtosis. Similarly, the association between the different factors and the outcomes was done by either the chi-square test for categorical factors or Student's *t*-test or Mann-Whitney *U* test for normally- and non-normally distributed continuous variables, respectively. DR incidence rate was calculated as the proportion (with 95% confidence interval) of patients with no DR at baseline who developed any DR at 5 years compared to that of all patients with no DR at baseline. DR worsening and progression rates were calculated as the proportions (with 95% confidence intervals) of patients with some DR at baseline who, respectively, worsened or improved at 5 years compared to all patients with DR at baseline. Stepwise multivariate logistic regression was carried on our two subgroups to identify factors associated with the incidence, worsening, and regression of DR. The *p* value for inclusion in the regression model was set at 0.1, whereas the *p* value for being retained in the model was 0.2.

## 3. Results

### 3.1. General Cohort Characteristics

Out of the 560 patients with type II diabetes mellitus who completed the initial evaluation, a total of 462 were able to be followed up on a yearly basis for a period of 5 years and are henceforth included in our analysis (Table 1). The 98 patients (17.50%) who were not included were lost to follow-up and could not be reached by their contact coordinates. Mean age at enrollment was 57.27 ± 10.91 years and 38.74% were males. Mean duration of disease at enrollment was 8.39 years with a standard deviation of 7.38 years and a range from less than 1 year to 44 years. HbA_1c_ had a mean value of 8.52 ± 2.06% and values ranging between 4.2% and 19.9%. Among these 462 patients, 281 (60.82%) had no DR at baseline. This group was looked at for the 5-year cumulative incidence of DR. The remaining 181 subjects had some form of DR in at least one eye at study entry, and only this group of patients was monitored for DR worsening or regression over a period of five years.

### 3.2. Diabetic Retinopathy Incidence and Risk Factors

At the end of the recruitment, there were 281 (60.82%) DR-free participants, with a mean age of 55.25 years. One hundred and eight of them were males (38.43%). This subset of patients had a mean duration of disease at enrollment of 5.51 years (range from less than 1 year to 20 years), and a mean HbA_1c_ of 8.34% (range from 4.2%–19.9%). After a total follow-up period of five years, 28 patients developed DR in at least one eye (10% DR incidence, 95% CI: 6–13). Twenty-two patients developed mild nonproliferative DR (NPDR) and remained as such until study closure, while six patients developed mild NPDR initially that later worsened to more advanced levels of DR. This group was not included in the worsening DR endpoint because of shorter follow-up duration as compared to the subgroup of patients with some DR at baseline. Bivariate analysis revealed only microalbuminuria and duration of disease to be significantly associated with the incidence of DR (*p* < 0.001 and *p* = 0.05, resp.), while HbA_1c_, systolic BP, diastolic BP, smoking, BMI, family history of diabetes, CAD, CVD, PVD, neuropathy, cholesterol, HDL, LDL, triglyceride levels, serum creatinine, and age at onset were not associated with the incidence of DR. Using stepwise logistic regression, only the presence of microalbuminuria at baseline strongly correlated with the development of DR (OR = 10.53, 95% CI: 4.39–25.23, *p* < 0.0001) (Table 2).

### 3.3. Diabetic Retinopathy Worsening and Risk Factors

One hundred and eighty-one patients (39.18%) presented with DR at the beginning of the study, having a mean age of 60.37 years (range 32–84), and 39.2% were male. Mean duration of disease at enrollment was 13 years (ranging from less than 1 year to 44 years), and mean HbA_1c_ was 8.8% (range from 4.7% to 15.1%). During the 5-year follow-up, 57 patients had their DR progress in at least one eye with a worsening rate of 31.5% (95% CI: 25–38) over 5 years. Fifty-seven eyes with preexisting mild NPDR (level 20) progressed by 2 grades to moderate NPDR (level 47), while five eyes with previously mild NPDR (level 20) progressed to PDR (level 53A). Eighteen eyes with moderate NPDR progressed to early PDR (level 61). Fourteen eyes with severe NPDR (level 53A) at baseline worsened to PDR (level 65 and above). Eight eyes labeled as early PDR (level 61) at study entry developed PDR with HRC or a diabetic tractional retinal detachment on follow-up (level 85). Stepwise logistic regression did not reveal any variable to be significantly associated with worsening of DR, but microalbuminuria approached statistical significance (OR = 1.89 95% CI: 0.97–3.70, *p* = 0.06) (Table 3).

### 3.4. Diabetic Retinopathy Regression and Risk Factors

After five years, 20 eyes of 16 (8.84%) patients out of the same cohort of 181 patients with initial DR showed improvement in their eye disease (9%, 95% CI: 5–13). Their mean age was 62.25 years and males consisted 62.50%. Analysis showed that the disease improved if the disease onset was at an older age (*p* = 0.004) and if the patient was male (*p* = 0.05). The patterns of improvement were as follows: 3 eyes improved from PDR to severe NPDR, whereas 2 eyes improved from PDR to moderate NPDR. 9 eyes improved from severe NPDR to moderate NPDR. Six eyes had their DR resolve: four of them had mild NPDR and two had moderate NPDR at baseline. Age at diagnosis of diabetes appeared to be the only significant independent predictor of the improvement of the disease in the logistic regression model, with OR = 1.07, 95% CI: 1.02–1.13, *p* = 0.009 (Table 4).

## 4. Discussion

To our knowledge, this is the first study from Lebanon that followed patients with type II diabetes over time for the development of or change in DR. Of the 462 patients who were monitored yearly for over 5 years by complete ophthalmic examination and fundus photographs, 281 had no DR at baseline and had a 10% cumulative 5-year incidence for the development of DR in at least one eye. Among the other 181 patients with some form of DR in at least one eye, the 5-year rates of worsening and regression were 31.5% and 9%, respectively.

In addition, risk factors for incidence of DR and progression and regression of the disease in patients with documented DR were looked at. While such data has been published in Caucasians (UKPDS [3], Blue Mountain [13], Wales [14], LALES [15], Liverpool Diabetic Eye Study [2], Central Australian Ocular Healthy Study [16]), and in the Indian subcontinent and the Far East (Beijing Eye Study [4]), there has yet to be such work from the Middle East.

The incidence of DR was linked to the presence of microalbuminuria and the duration of disease. While this result is somewhat in agreement with other prospective large-scale studies such as the WESDR [5, 6], it approaches more hospital-based studies of similar scale and methodology [17]. However, unlike other studies, poor glycemic control was not associated with the development of DR. This is possibly due to the fact that our subgroup of patients with no initial DR at study entry had a relatively good glycemic control (HbA_1c_ = 7.4%), which was significantly lower than the HbA_1c_ of the subgroup with some DR at baseline (HbA_1c_ = 8.78%) (*p* < 0.0001). Furthermore, the former subgroup was recently diagnosed (62.1% had a duration of disease of 5 years or less), giving them access to newer therapies, which lead them to maintain good glycemic control throughout their five-year follow-up (HbA_1c_ = 7.2%). Moreover, the number of patients who develop some DR during their 5-year follow-up is low, which may affect the statistical analysis.

DR worsening on the other hand had only microalbuminuria associated with disease progression, with values close to statistical significance (OR = 1.89, 95% CI: 0.97–3.70, *p* = 0.06). Other classic risk factors such as duration of diabetes and age at diagnosis were not reproduced in our cohort. One possible explanation to this is the fact that our criteria for worsening were more stringent than other studies. Perhaps setting worsening criteria as an increase of one level of DR instead of two could alter the associations of classic risk factors with worsening of DR. However, an increase of two levels of DR might be more clinically relevant. Finally, control of diabetes has improved with time; in fact, our subgroup from the cohort started with an HbA_1c_ that was relatively elevated and improved albeit mildly over time.

Spontaneous DR regression was rare, which is in agreement with other studies. Moreover, most patients who experienced some regression only improved by one level of DR. Weak associations with regression were late age at onset and negative family history of DM, both of which suggest a short duration of disease and possibly a weak genetic factor.

Our study has some limitations that ought to be addressed. First, as a hospital-based population, our numbers might be different compared to community-based studies. Patients presenting to a diabetes clinic generally have better control and knowledge of their disease than patients not being followed up rigorously [18, 19]. In addition, a larger number of patients with a longer follow-up period might reveal different results.

## 5. Conclusion

In summary, we present the results of a hospital-based cohort of patients with type II diabetes with suboptimal control, strong family history, and important coexisting complications followed up over a period of 5 years with a relatively low incidence of DR development (10%), despite a high local prevalence of diabetes. Microalbuminuria was an independent risk factor associated with the incidence of the disease and its progression to more severe stages. It is therefore our recommendation to insist on the screening for microalbuminuria in patients with type II diabetes as a prognostic sign for the development DR in patients with no DR at baseline.

## Figures and Tables

**Table 1:** Baseline characteristics of the study cohort.

Total sample	*n* = 462
Age (years), mean ± SD	57.3 ± 10.9
Male, *n* (%)	179 (38.74)
Age at diagnosis (years), mean ± SD	49.36 ± 10.76
Duration of diabetes (years), median (IQR)	6.00 (9.00)
Family history of diabetes, *n* (%)	334 (72.29)
Consanguineous parents, *n* (%)	100 (21.65)
Current smoker, *n* (%)	141 (30.52)
Smoking (pack-years), median (IQR)	22.00 (25.00)
Coronary artery disease, *n* (%)	108 (23.37)
Cerebrovascular disease, *n* (%)	20 (4.33)
Peripheral vascular disease, *n* (%)	86 (18.61)
Neuropathy, *n* (%)	173 (37.45)
Body mass index (kg/m^2^), mean ± SD	30.41 ± 5.62
HbA_1c_, *n* (%)	8.52 ± 2.06
Serum creatinine (mg/dL), median (IQR)	0.80 (0.30)
Urine microalbuminuria, positive, *n* (%)	160 (34.63)
Urine macroalbuminuria, positive, *n* (%)	50 (10.82)
Systolic blood pressure (mmHg), mean ± SD	129.24 ± 16.15
Diastolic blood pressure (mmHg), mean ± SD	79.59 ± 9.57
Duration of hypertension (years), median (IQR)	1.00 (5.00)
Cholesterol (mg/dL), mean ± SD	201.22 ± 49.70
HDL (mg/dL), mean ± SD	44.22 ± 13.58
LDL (mg/dL), mean ± SD	118.72 ± 37.37
Triglycerides (mg/dL), median (IQR)	156.00 (107.00)

**Table 2:** Characteristics of the patients with no diabetic retinopathy at baseline according to the development or not of retinopathy.

	No baseline DR (*n* = 253)	Incident DR (*n* = 28)	*p* value
Age (years), mean ± SD	55.47 ± 11.52	53.25 ± 8.75	0.32
Male, *n* (%)	96 (37.94)	12 (42.86)	0.61
Age at diagnosis (years), mean ± SD	50.74 ± 11.15	47.93 ± 8.83	0.25
Duration of diabetes (years), median (IQR)	4.00 (6.00)	6.50 (6.00)	0.05
Family history of diabetes, *n* (%)	173 (68.38)	20 (71.43)	0.74
Consanguineous parents, *n* (%)	60 (23.72)	3 (10.71)	0.13
Current smoker, *n* (%)	86 (33.99)	10 (35.71)	0.96
Smoking (pack-years), median (IQR)	25.00 (30.00)	25.00 (25.00)	0.95
Coronary artery disease, *n* (%)	46 (18.18)	3 (10.71)	0.44
Cerebrovascular disease, *n* (%)	5 (1.97)	1 (3.57)	0.47
Peripheral vascular disease, *n* (%)	30 (11.86)	1 (3.57)	0.34
Neuropathy, *n* (%)	75 (29.64)	8 (28.57)	0.91
Body mass index (kg/m^2^), mean ± SD	30.64 ± 5.90	29.63 ± 3.62	0.64
HbA_1c_, *n* (%)	8.27 ± 2.09	8.98 ± 2.48	0.10
Serum creatinine (mg/dL), median (IQR)	0.80 (0.20)	0.80 (0.20)	0.86
Urine microalbuminuria, positive, *n* (%)	46 (18.18)	19 (67.86)	<0.0001
Urine macroalbuminuria, positive, *n* (%)	9 (3.56)	3 (10.71)	0.09
Systolic blood pressure (mmHg), mean ± SD	125.99 ± 15.69	128.75 ± 11.52	0.14
Diastolic blood pressure (mmHg), mean ± SD	78.18 ± 8.99	80.32 ± 8.79	0.23
Duration of hypertension (years), median (IQR)	0.00 (4.00)	0.00 (5.00)	0.72
Cholesterol (mg/dL), mean ± SD	203.93 ± 48.85	192.71 ± 47.84	0.27
HDL (mg/dL), mean ± SD	44.85 ± 13.43	47.71 ± 17.63	0.40
LDL (mg/dL), mean ± SD	119.63 ± 39.27	110.79 ± 42.22	0.21
Triglycerides (mg/dL), median (IQR)	150.00 (102.00)	180.00 (120.00)	0.68
	^∗^OR (95% CI)		
Urine microalbuminuria, positive	10.53 (4.39–25.23)		<0.0001
Cholesterol	0.99 (0.98–1.00)		0.09

DR: diabetic retinopathy. ^∗^Odds ratio for the predictors of development of DR in patients with type II diabetes but no DR at baseline obtained using multivariate logistic regression analysis, with variables—urine microalbuminuria, urine macroalbuminuria, HbA_1c_, serum creatinine, duration of diabetes, age at diagnosis, duration of hypertension, cholesterol, HDL, LDL, triglycerides, family history of diabetes, and body mass index.

**Table 3:** Characteristics of the patients with diabetic retinopathy at baseline according to the worsening or not of their retinopathy.

	No worsening DR (*n* = 124)	Worsening DR (*n* = 57)	*p* value
Age (years), mean ± SD	60.25 ± 9.91	60.60 ± 8.80	0.81
Male, *n* (%)	48 (38.71)	23 (40.35)	0.83
Age at diagnosis (years), mean ± SD	47.57 ± 10.18	47.47 ± 10.18	0.95
Duration of diabetes (years), median (IQR)	12.00 (14.00)	10.00 (8.00)	0.92
Family history of diabetes, *n* (%)	95 (76.61)	46 (80.70)	0.54
Consanguineous parents, *n* (%)	25 (20.16)	12 (21.05)	0.89
Current smoker, *n* (%)	29 (23.39)	15 (26.32)	0.19
Smoking (pack-years), median (IQR)	17.50 (25.00)	28.50 (28.00)	0.13
Coronary artery disease, *n* (%)	40 (32.26)	20 (35.09)	0.71
Cerebrovascular disease, *n* (%)	8 (6.45)	7 (12.28)	0.25
Peripheral vascular disease, *n* (%)	38 (30.65)	18 (31.58)	0.90
Neuropathy, *n* (%)	63 (50.81)	28 (49.12)	0.83
Body mass index (kg/m^2^), mean ± SD	30.60 ± 5.97	29.39 ± 4.23	0.12
HbA_1c_, *n* (%)	8.64 ± 1.85	9.08 ± 2.07	0.15
Serum creatinine (mg/dL), median (IQR)	0.90 (0.49)	0.90 (0.50)	0.73
Urine microalbuminuria, positive, *n* (%)	61 (49.19)	34 (59.65)	0.21
Urine macroalbuminuria, positive, *n* (%)	27 (21.77)	12 (21.05)	0.91
Systolic blood pressure (mmHg), mean ± SD	134.20 ± 16.17	133.23 ± 16.98	0.71
Diastolic blood pressure (mmHg), mean ± SD	81.61 ± 10.03	80.96 ± 10.54	0.69
Duration of hypertension (years), median (IQR)	2.00 (10.00)	4.00 (10.00)	0.55
Cholesterol (mg/dL), mean ± SD	197.80 ± 43.28	201.14 ± 65.59	0.69
HDL (mg/dL), mean ± SD	42.10 ± 11.99	44.11 ± 14.98	0.38
LDL (mg/dL), mean ± SD	119.99 ± 36.80	116.11 ± 26.56	0.48
Triglycerides (mg/dL), median (IQR)	174.50 (112.00)	144.00 (114.00)	0.24
	^∗^OR (95% CI)		
Urine microalbuminuria, positive	1.89 (0.97–3.70)		0.06
Serum creatinine	0.56 (0.29–1.07)		0.08
Body mass index	0.96 (0.90–1.02)		0.14

DR: diabetic retinopathy. ^∗^Odds ratio for the predictors of worsening of DR in patients with type II diabetes and DR at baseline obtained using multivariate logistic regression analysis, with variables—urine microalbuminuria, urine macroalbuminuria, HbA_1c_, serum creatinine, duration of diabetes, age at diagnosis, duration of hypertension, cholesterol, HDL, LDL, triglycerides, family history of diabetes, and body mass index.

**Table 4:** Characteristics of the patients with diabetic retinopathy at baseline according to the regression or not of their retinopathy.

	No regressed DR (*n* = 165)	Regressed DR (*n* = 16)	*p* value
Age (years), mean ± SD	60.18 ± 9.72	62.25 ± 7.61	0.32
Male, *n* (%)	61 (36.97)	10 (62.50)	0.05
Age at diagnosis (years), mean ± SD	46.90 ± 10.19	54.13 ± 7.12	0.004
Duration of diabetes (years), median (IQR)	11.00 (13.00)	8.50 (12.00)	0.10
Family history of diabetes, *n* (%)	131 (79.39)	10 (62.50)	0.13
Consanguineous parents, *n* (%)	34 (20.61)	3 (18.75)	1.00
Current smoker, *n* (%)	40 (24.24)	4 (25.00)	1.00
Smoking (pack-years), median (IQR)	20.00 (23.00)	35.00 (29.00)	0.35
Coronary artery disease, *n* (%)	54 (32.73)	6 (37.50)	0.70
Cerebrovascular disease, *n* (%)	15 (9.09)	0 (0.00)	0.37
Peripheral vascular disease, n (%)	54 (32.73)	2 (12.50)	0.15
Neuropathy, *n* (%)	85 (51.52)	6 (37.50)	0.28
Body mass index (kg/m^2^), mean ± SD	30.33 ± 5.56	29.01 ± 4.80	0.37
HbA_1c_, *n* (%)	8.84 ± 1.88	8.20 ± 2.34	0.21
Serum creatinine (mg/dL), median (IQR)	0.90 (0.50)	1.05 (0.58)	0.34
Urine microalbuminuria, positive, *n* (%)	85 (51.52)	10 (62.50)	0.41
Urine macroalbuminuria, positive, *n* (%)	35 (21.21)	4 (25.00)	0.75
Systolic blood pressure (mmHg), mean ± SD	133.91 ± 15.93	133.75 ± 21.10	0.83
Diastolic blood pressure (mmHg), mean ± SD	80.94 ± 9.73	86.25 ± 13.35	0.15
Duration of hypertension (years), median (IQR)	4.00 (10.00)	1.00 (7.00)	0.25
Cholesterol (mg/dL), mean ± SD	200.08 ± 52.32	186.13 ± 37.29	0.34
HDL (mg/dL), mean ± SD	42.90 ± 13.45	41.00 ± 6.68	0.87
LDL (mg/dL), mean ± SD	119.51 ± 34.09	111.19 ± 31.71	0.35
Triglycerides (mg/dL), median (IQR)	166.00 (115.00)	162.00 (73.00)	0.96
	^∗^OR (95% CI)		
Age at diagnosis	1.07 (1.02–1.13)		0.009
Serum creatinine	1.30 (0.90–1.89)		0.17

DR: diabetic retinopathy. ^∗^Odds ratio for the predictors of regression of DR in patients with type II diabetes and DR at baseline obtained using multivariate logistic regression analysis, with variables—urine microalbuminuria, urine macroalbuminuria, HbA_1c_, serum creatinine, duration of diabetes, age at diagnosis, duration of hypertension, cholesterol, HDL, LDL, triglycerides, family history of diabetes, and body mass index.
