# Corrigendum to “Five-Year Incidence and Progression of Diabetic Retinopathy in Patients with Type II Diabetes in a Tertiary Care Center in Lebanon”

**DOI:** 10.1155/2018/2424757

**Published:** 2018-07-22

**Authors:** Carl-Joe Mehanna, Maamoun Abdul Fattah, Hani Tamim, Mona P. Nasrallah, Raya Zreik, Sandra S. Haddad, Jaafar El-Annan, Samih Raad, Randa S. Haddad, Haytham I. S. Salti

**Affiliations:** ^1^Department of Ophthalmology, American University of Beirut Medical Center, Beirut, Lebanon; ^2^Biostatistics Unit, Department of Internal Medicine, American University of Beirut Medical Center, Beirut, Lebanon; ^3^Division of Endocrinology and Metabolism, Department of Internal Medicine, American University of Beirut Medical Center, Beirut, Lebanon; ^4^Fouad Khoury Hospital Bikhazi Medical Group, Beirut, Lebanon; ^5^Department of Ophthalmology, University of Texas Medical Branch, Galveston, TX, USA; ^6^Fellow-Division of Pulmonary, Critical Care & Sleep Medicine, University of Oklahoma Health Sciences Center, Oklahoma City, OK, USA

In the article titled “Five-Year Incidence and Progression of Diabetic Retinopathy in Patients with Type II Diabetes in a Tertiary Care Center in Lebanon” [1], Dr. Samih Raad was missing from the authors' list. The corrected authors' list and affiliations are shown above and updated in place.

Additionally, the following Acknowledgements section should be added:

“The authors wish to thank Spephal Group Lebanon for their continuous and generous support.”

The Conflicts of Interest section also should be changed from “None of the authors have any proprietary interests or conflicts of interest related to this submission” to “Spephal Group Lebanon, a company who markets eye care treatments, sponsored the publication of the article.”

These corrections are made in place.
